# Clinical assessment, neuroimaging and immunomarkers in Chagas disease study (CLINICS): Rationale, study design and preliminary findings

**DOI:** 10.1590/S1980-57642012DN06030012

**Published:** 2012

**Authors:** Jamary Oliveira-Filho, Jesângeli de S. Dias, Pedro A.P. Jesus, Nestor J.S.B. Neto, Roque Aras, Francisco J.F.B. Reis, Karen L. Furie

**Affiliations:** From the Stroke and Cardiomyopathy Clinics of the Hospital Universitario Professor Edgard Santos, Federal University of Bahia, BA, Brazil; and the Stroke Service of Massachusetts General Hospital, Harvard University, USA.

**Keywords:** Chagas disease, stroke, cognitive impairment, brain atrophy, biomarkers

## Abstract

Chagas disease (CD) is an important cause of cardiomyopathy and stroke in Brazil. Brain infarcts and atrophy seem to occur independently of cardiomyopathy severity and cognitive impairment is understudied.

**Objective:**

Compare the prevalence of brain magnetic resonance imaging abnormalities between patients with or without CD; determine if inflammatory biomarkers are increased in CD; and determine the efficacy of aspirin in reducing the rate of microembolization in these patients.

**Methods:**

500 consecutive patients with heart failure will undergo a structured cognitive evaluation, biomarker collection and search for microembolic signals on transcranial Doppler. The first 90 patients are described, evaluated with cognitive tests and brain magnetic resonance imaging to measure N-acetyl aspartate (NAA), choline (Cho), myo-inositol (MI) and creatine (Cr).

**Results:**

Mean age was 55±11 years, 51% female, 38 (42%) with CD. Mean NAA/Cr ratio was lower in patients with CD as compared to other cardiomyopathies. Long-term memory and clock-drawing test were also significantly worse in CD patients. In the multivariable analysis correcting for ejection fraction, age, sex and educational level, reduced NAA/Cr (p=0.006) and cognitive dysfunction (long-term memory, p=0.023; clock-drawing test, p=0.015) remained associated with CD.

**Conclusion:**

In this preliminary sample, CD was associated with cognitive impairment and decreased NAA/Cr independently of cardiac function or educational level.

## Abstract (pt)

A doença de Chagas (DC) é causa importante de cardiomiopatia e acidente vascular cerebral no Brasil. Os infartos e atrofia cerebral na DC parecem ocorrer independente da gravidade da cardiomiopatia, sendo o comprometimento cognitivo pouco estudado.

**Objetivo:**

Determinar a prevalência de alterações na ressonância magnética entre chagásicos e não chagásicos; determinar se os níveis de marcadores inflamatórios estão aumentados na DC e determinar a eficácia da aspirina em reduzir a taxa de microembolização nestes pacientes.

**Métodos:**

Quinhentos pacientes consecutivos com diagnóstico de insuficiência cardíaca serão submetidos a uma avaliação cognitiva estruturada, coleta de biomarcadores e pesquisa de sinais de microembolia por Doppler transcraniano. Os primeiros 90 pacientes são descritos, avaliados por testes cognitivos e ressonância magnética cerebral, com medida de N-acetil aspartato (NAA), colina (Cho), mioinositol (MI) e creatina (Cr).

**Resultados:**

A idade média foi de 55±11 anos, 51% eram do sexo feminino, 38 (42%) tinha DC. A média da relação NAA/Cr foi mais baixa em pacientes com DC quando comparada com outras miocardiopatias. O desempenho nos testes de memória de longo prazo e desenho do relógio foi significativamente pior nos portadores de DC. Na análise multivariada, corrigindo para fração de ejeção, idade, gênero e nível educacional, redução da relação NAA/Cr (p=0.006) e disfunção cognitiva (memória de longo prazo, p=0.023; teste do desenho do relógio, p=0.015) permaneceram associados a DC.

**Conclusão:**

Nesta amostra preliminar, a doença de Chagas esteve associada a disfunção cognitiva e redução dos níveis de NAA/Cr, independente da função cardíaca e nível educacional.

## INTRODUCTION

Chagas disease (CD), also known as American trypanosomiasis, is caused by a flagellated protozoan named *Trypanosoma cruzi*, first described in 1911 by Carlos Chagas.^1^ Although more than a century has elapsed since its initial description, Chagas disease remains a major public health problem. According to the World Health Organization (WHO), there are 10 million people infected worldwide, most of whom are living in Latin America, where the disease is endemic.^2^ However, cases have been described in North America and Europe, mostly associated with transfusion of infected blood from Latin American immigrants.^2-4^. In its symptomatic chronic form, CD primarily affects the gastrointestinal tract and heart, representing an important cause of heart disease in Latin America. Around 30% of infected individuals will develop chronic chagasic cardiomyopathy (CCC), which is manifested as heart failure (HF), conduction disorders, arrhythmias, sudden death and thromboembolic phenomena.^5^

Stroke in patients with CD was first described by Nussenzweig et al. in 1953.^6^ Since then, there have been numerous publications on the subject.^7-14^ In retrospective studies of autopsies, cerebral infarction was found in 10%-35% of cases^8,9,15^. The brain hemispheres are the most affected areas, especially in the region of the parietal, temporal and frontal lobes, as well as the basal ganglia. Old infarcts, cortical laminar necrosis and cerebral atrophy are other pathological findings observed in these studies^8,9,15^. Some studies have analyzed the risk of stroke in patients with CD. A case-control study performed in Colombia found a higher frequency of *T. cruzi* infection among patients with stroke than in controls without stroke (24.4% *vs.* 1.9%).^11^ In a cross-sectional study among patients with cardiomyopathy, stroke was more frequent among patients with CCC, compared to other types of cardiomyopathy (OC) (15% *vs.* 9.3%, p=0.01).^12^ Another study found that CD patients, with or without cardiomyopathy, have a three times greater risk of stroke compared to controls of the same sex and age without CD.^16^ Regarding the mechanism of stroke in patients with CD, the embolic etiology seems clear - thrombi associated with systolic dysfunction and aneurysm of the tip of the left ventricle, as well as mural thrombi, have been described in these patients (by apud Oliveira JSM).^13,17^ However, this does not appear to be the only mechanism. Other studies show that having CD is an independent risk factor for stroke, even when adjusted for potential confounders.^12^ In a population of stroke patients, 37% of those who had undetermined etiology for the event had positive serology for CD.^10^ Recently, ischemic stroke was reported as the first manifestation of CD, even in the absence of cardiac arrhythmia and left ventricle dysfunction.^18^ Jesus et al., searching for signs of microemboli on transcranial Doppler in a cardiomyopathy clinic, showed that microemboli are more frequent among patients with CCC compared to patients with other heart diseases, independent of left ventricle function, and are also associated with silent heart attacks.^19^ Silent brain infarcts have also been described by another author.^10^ It is speculated that inflammation^18^ and thrombogenic factors^20^ play a role in this mechanism, but these need to be further clarified.

Cognitive impairment in Chagas disease is a controversial and poorly explored subject. Mangone et al.^21^ studied the performance on cognitive tests of 45 asymptomatic patients with chronic CD compared to that of 20 normal controls matched for age, educational level and years living in endemic areas. Results revealed poor performance on nonverbal reasoning, orientation, sequencing and problem solving, as well as difficulty encoding new information. The authors speculated that the neuropsychological findings resemble the cognitive changes observed in diseases of cerebral white matter. Dias et al.^22^ studied 37 patients with CCC and 42 patients with other cardiomyopathies (OC) matched for gender, educational level and cardiac systolic function, comparing them using the following tests: Mini-Mental State Examination (MMSE), Brief Cognitive Screening Battery, Digit Span Test (forward and backward sequences), Rey Auditory-Verbal Learning Test, Rey Osterreith Complex Figure test (ROCF) and the Hospital Anxiety and Depression Scale. Overall, cognitive performance of the two groups was similar, except for the late recall of the Rey Osterreith Complex figure, where even after correcting for potential confounders (age, education, functional class of HF, use of medications), the performance of the Chagas disease group was significantly worse. Smid et al.^23^ analyzed the clinical characteristics of an outpatient sample of patients with vascular dementia, and found that 8% of the participants had CD. A population-based study conducted among elderly people in Bambuí, an endemic region of Minas Gerais for CD, showed an important association between *T. cruzi* infection and low scores on the MMSE, even after correcting for potential confounders.^24^ Other authors tried to correlate the presence of hyperintense white matter lesions seen on magnetic resonance imaging of the skull (cranial MRI)^25^ with performance on cognitive tests, but attributed the changes found to the mere effect of low education. Wackermann et al., despite having observed changes in the EEG of these patients, as well as signs of involvement of white matter on brain MRI, found no significant clinical repercussions in these patients.^26^ Despite the absence of an anatomical-pathological basis for possible chronic brain involvement of CD, and likewise for the heart and digestive tract, as initially thought by Carlos Chagas^8,9^ the association between cognitive impairment and CD is biologically plausible. Severe heart failure and thromboembolic complications are common in patients with CD. Studies have shown that CD is an important risk factor for stroke in our region,^10,12,18^ and that cerebral infarcts lead to vascular cognitive impairment.^27^ Furthermore, brain atrophy in these patients has been described in 3.2 to 15,7% of cases,^8,9^ but the authors ascribed this phenomenon to hypoxic-ischemic events of heart failure, which appears to correlate with its severity. HF is associated with cognitive impairment.^28,29^ However, in a study of the brain through cranial CT scan involving a sample of patients with heart disease, it was shown that the presence of brain atrophy was independently associated with Chagas disease, even after correcting for degree of myocardial dysfunction on echocardiography.^30^ The authors speculated that this finding may be related to elevated levels of inflammatory markers.

## CLINICS

In order to further investigate this important disease we designed the "Clinical assessment, neuroimaging and immunomarkers in Chagas disease study" (CLINICS). CLINICS is an NIH and CNPq-funded study involving a collaboration between two outpatient clinics at the Federal University of Bahia (Stroke and Cardiomyopathy) and the Stroke Service at Massachusetts General Hospital, Harvard University, with an estimated sample size of 500 patients and registered as a clinical trial under www.clinicaltrials.gov (identifier NCT01650792). In CLINICS, we will address three specific aims and corresponding hypotheses:

**Specific Aim 1.** To determine the prevalence of high-risk brain magnetic resonance imaging characteristics in patients with Chagasic CM compared with non-Chagasic CM.

*Hypothesis #1:* Silent infarcts detected on magnetic resonance imaging will be more common in patients with Chagasic CM as compared to other CM etiologies (e.g., ischemic, idiopathic).

*Hypothesis #2:* Brain atrophy will be more common in patients with Chagasic CM as compared to other CM etiologies (e.g., ischemic, idiopathic).

*Hypothesis #3:* Volume of white matter hyperintensity will be greater in patients with Chagasic CM as compared to other CM etiologies (e.g., ischemic, idiopathic).

**Specific Aim 2.** To determine whether levels of inflammatory and peptide degrading biomarkers are increased in Chagasic CM.

*Hypothesis #1:* Mean levels of orosomucoid, interleukin-6 (IL-6), metalloproteinase-9, and neprilysin will be higher in patients with Chagasic CM as compared to other CM etiologies (e.g., ischemic, idiopathic).

*Hypothesis #2:* Mean levels of orosomucoid, IL-6, metalloproteinase-9, and neprilysin will predict silent infarcts, white matter hyperintensities, and high-intensity transient signals (HITS) in patients with Chagasic CM.

**Specific Aim 3.** To evaluate the efficacy of aspirin therapy in decreasing microembolization rate in patients with Chagasic CM.

*Hypothesis #1:* High-intensity transient signals (HITS) detected on transcranial Doppler will be more common in patients with Chagasic CM as compared to other CM etiologies (e.g., ischemic, idiopathic).

*Hypothesis #2:* The proportion of Chagas patients with HITS will decrease after 1 week of treatment with aspirin.

*Hypothesis #3:* Levels of orosomucoid, IL-6, metalloproteinase-9, and neprilysin will decrease with aspirin therapy.

**Neuroimaging markers of stroke risk and cognitive impairment**. Several markers of stroke risk have been studied on both brain CT and MRI. The most studied markers include the presence of silent infarcts, white matter hyperintensity and brain atrophy. The significance of silent infarcts was extensively studied in 3,324 patients at risk for stroke who underwent brain MRI in the Cardiovascular Health Study.^31^ Overall, 28% of these patients had silent infarcts at baseline, which were independently associated with future stroke risk at a 4-year follow-up. Patients with silent infarcts do not typically receive the same type of medical intervention for secondary prophylaxis as patients presenting with a clinically-evident stroke. Thus, these patients might be at a particularly high risk of stroke recurrence.

Another study from the same group investigated the role of white matter hyperintensity on MRI.^32^ MRI scans were performed twice over 5 years in 1,919 patients at risk for stroke. White matter hyperintensity progression was found to correlate with cognitive worsening over time, tested by serial Mini-Mental State Examination and Digit-Symbol Substitution Test. Hachinski coined the term "leuko-araiosis" to denote the subcortical and periventricular white-matter hypointensity seen on brain CT.^33^ Although probably heterogeneous in pathophysiology, leuko-araiosis has been related to risk of future stroke and cognitive decline.

Brain atrophy has been most extensively studied in patients with mild cognitive impairment, vascular dementia and Alzheimer's disease. Both CT and MRI studies have correlated regional (e.g., temporal lobe) or global brain volumes with degree of cognitive impairment. Indices such as ventricle-to-brain ratio were also demonstrated to be useful in patients with Alzheimer's disease. Recently, a substudy from the Framingham cohort demonstrated that chronic inflammation (increase in TNF-alpha and IL-6) was associated with brain atrophy but not white matter hyperintensity.^34^ Thus, chronic inflammation may be a link between Chagas disease and brain involvement not only via brain infarction, but also through progressive brain atrophy.

**High Intensity Transient Signals (HITS) on transcranial Doppler in ischemic stroke.** Since 1982, transcranial Doppler (TCD) has been used as a non-invasive means to study the intracranial vessels.^35^ One of the unique abilities of TCD is that it allows real-time monitoring of intracranial vessels for the occurrence of high intensity transient signals (HITS). During heart or carotid surgery, HITS may be due to solid as well as gaseous material. In outpatients, however, HITS are most likely a reflection of silent thromboembolism in high-risk patients. Experimental studies have demonstrated that TCD is highly sensitive and specific for detecting platelet or thrombus emboli.^36^ These occur more frequently during the acute phase of ischemic stroke^37^ and their presence indicates a higher risk of future stroke.^38^ In one study, 17% of patients referred for echocardiography presented with HITS, while high-risk sources for emboli such as metallic valve prosthesis showed HITS in 33% of cases.^39^ One study used HITS as a surrogate endpoint for evaluating efficacy of two different antiplatelet regimens for patients with carotid stenosis and recent stroke.^40^ With a smaller sample size than would be required to demonstrate clinical efficacy, patients receiving the combination of aspirin and clopidogrel were 40% less likely to have HITS after 7 days of treatment as compared to patients on monotherapy with aspirin.^40^

Patients with high-risk sources for emboli such as atrial fibrillation showed a 15% proportion of HITS.^39^ In comparison, our preliminary data shows that 16% of patients with Chagas disease have HITS, which is significantly higher than a control population with other cardiomyopathies (2%). Furthermore, patients with HITS were at an increased risk of death on follow-up. Taken together, these data suggest that HITS may be a useful noninvasive marker of embolic risk in patients with Chagas disease and could be used as a surrogate endpoint for testing the efficacy of new drugs.

**Chronic inflammation and endopeptidases in ischemic stroke.** The chronic manifestations of Chagas disease appear to be immune-mediated and, based on our preliminary data, are associated with increased levels of inflammation and activation of endopeptidases. These secondary systemic responses may have profound effects on the central nervous system, increasing the risk of vascular injury and dementia.

*Interleukin-6 (IL-6).* In cardiovascular disease, C-reactive protein (CRP), a marker of inflammation, and fibrinogen are highly correlated.^41^ Inflammation and coagulation interact in patients with conventional vascular risk factors. In hypercholesterolemic patients, factor VII clotting activity, FVII antigen and activated FVII levels were found to correlate with CRP, interleukin-6 soluble receptor, P-selectin, soluble intercellular adhesion molecule-1 and transforming growth factor-beta.^42^ FVII levels were positively associated with CRP and IL-6 soluble receptor.^43^ In patients with renal insufficiency, CRP, fibrinogen, factor VII, factor VIII, IL-6, intercellular adhesion molecule-1, D-dimer, and PAP levels were intercorrelated. The strongest correlations were among CRP, IL-6, and fibrinogen.^44^

Plasma IL-6 concentrations have been correlated with CT brain infarct volume (r=0.75) and mRS at 3 months (r=0.72).^45^ IL-6 levels were also strongly associated with CRP levels and white blood cell (WBC) count.^45^ In the Framingham Offspring Study, inflammatory biomarkers were associated with neuroimaging markers of ischemia and dementia (e.g. white matter hyperintensities and total brain volume). CD40 ligand, C-reactive protein, interleukin-6 (IL-6), soluble intracellular adhesion molecule-1, monocyte chemoattractant protein-1, myeloperoxidase, osteoprotegerin (OPG), P-selectin, tumor necrosis factor-alpha (TNF-alpha), and tumor necrosis factor receptor II were measured on 1926 subjects. On multivariable models, inflammatory markers were associated with total brain volume (p<0.0001) but not white matter hyperintensities. IL-6 levels were inversely associated with brain volume.^34^

*Orosomucoid*. Orosomucoid, or a1 glycoprotein, is a marker of inflammation. Inflammation appears to increase the risk associated with conventional vascular risk factors. A study examining five inflammation-sensitive plasma proteins (fibrinogen, alpha1-antitrypsin, haptoglobin, ceruloplasmin, and orosomucoid) in 6063 healthy men, aged 28 to 61 years, found that after risk factor adjustment, men with hypercholesterolemia and high inflammatory marker levels, but not those with hypercholesterolemia alone, had a significantly higher risk of ischemic stroke (RR=2.1; CI, 1.4 to 3.3).^46^ Similarly, high levels of inflammatory markers increased the risk of stroke in diabetics (risk factor-adjusted relative risk 2.5 (1.4-4.6)) and smokers.^41,47^

*Matrix metalloproteinase-9 (MMP-9)*. MMP9 (gelatinase B), one of more than 20 zinc-dependent endopeptidases that participate in extracellular matrix remodeling, has been implicated as a major mediator of early BBB disruption in animal models of spontaneous post-stroke intracerebral hemorrhage (ICH).^43,44,48-52^ Conversely, inhibition of MMP^9^ results in decreased infarct volume and decreased rate of hemorrhagic conversion.^53-55^ In a murine model of MMP-deficiency, low expression of MMP9 protected against BBB injury and resulted better motor recovery versus controls.^56,57^ In addition, clinical data in humans also support a correlation between peak levels of MMP9, severity of stroke and development of hemorrhagic conversion following ischemic stroke. Data indicate that plasmin, MMP9 and MMP2 mediate basal lamina and endothelial injury, which may accelerate ischemic edema and ICH formation.^58,59^ Clinical trial data indicate that the risk of hemorrhage is greatly increased in areas of early brain edema on CT, particularly as the time from onset to treatment increases.^60^ Following brain embolism, the usual natural history is spontaneous dissolution of the embolic fragment within the first 72 hours.^61-63^ This leads to reperfusion of the ischemic microvascular bed, and frequently to edema and hemorrhagic transformation (HT), varying from petechiae to large parenchymal hematoma (PH).^62,63^ MMP9 appears to mediate early opening of the BBB, with peaks at 15 hours after temporary middle cerebral artery occlusion (MCAO), compared to a late increase in gelatinase A (MMP2) at 5 days.^64^ Clinically, severe edema and ICH are major causes of secondary brain injury, early death, and post-stroke disability.^60-66^ Severe edema is common after large middle cerebral artery (MCA) infarcts, where it is the commonest cause of early neurological death due to brain herniation and brainstem compression (mortality 5%-45%).^65,66^ Post-ischemic cortical spreading depression has been shown to induce blood-brain barrier disruption which is mediated through MMP-9. Increased MMP-9 expression has also been associated with unstable carotid atherosclerotic plaques.^67^ No studies to date have investigated the role of MMPs in Chagas disease models, but preliminary data from our group has shown that MMP-9 gene expression is increased in Chagas disease patients.

Inflammation is an important alternative pathway of MMP activation and a potential confounder in analyzing the association between oxidative stress and MMPs. Interleukin-1β, a cytokine synthesized by endothelium, microglia, astrocytes, and neurons has proinflammatory, procoagulant, and cell-growth functions.^68^ IL-1b expression increases early (within 2 hours) in focal ischemia.^69-72^ Exogenous application of recombinant human interleukin-1β into the ventricle immediately after reperfusion resulted in increased edema, infarct size, number of infiltrating neutrophils, and number of endothelial-bound neutrophils.^73^ As an upstream inflammatory stimulant of MMP expression, the increase in edema associated with IL-1β may be MMP-mediated.^74^

*Neprilysin (NEP)*. Chagas disease and Alzheimer's disease are both associated with brain atrophy, although the mechanism in Chagas has not been well-defined. Alzheimer's disease (AD) is characterized by accumulation of extracellular deposits of A beta. AD has been linked to vascular risk factors, inflammation, and ischemic stroke. Neprilysin (NEP) is a zinc metalloproteinase expressed in the brain which serves as an amyloid beta-peptide (Abeta) degrading enzyme. NEP is part of an endogenous mechanism of Abeta removal via proteolytic degradation.^75^ Beta-amyloid levels are significantly elevated in neprilysin knock-out mice, and NEP inhibitors cause a rapid increase in beta-amyloid concentrations.^76^ Although speculative, it could be the case that levels of neprilysin are increased in response to increased Abeta deposition, secondary to elevated levels of inflammation. Furthermore, NEP is also involved in angiotensin metabolism, converting vasocontricting peptide angiotensin I to a vasodilating peptide, angiotensin 1-7.^77,78^ NEP has been studied as a therapeutic target, with dual ACE/NEP inhibition decreasing cardiac and vascular fibrosis in spontaneously hypertensive rats.^79^

We will be studying 500 patients, >18 years of age, with congestive heart failure and no previous history of stroke. Congestive heart failure will be defined according to clinical criteria composed of signs (progressive lower extremity edema and hepatomegaly not attributable to other systemic diseases) and symptoms (exercise-induced dyspnea and paroxysmal nocturnal dyspnea), classified according to New York Heart Association functional classes. Additionally, patients with Chagas disease will require typical findings on either echocardiogram (inferior wall motion abnormalities, apical aneurysm or thrombus) or electrocardiogram (right bundle branch or atrioventricular block), according to the Brazilian Consensus in Chagas Disease.^80^ Cases and controls will be matched 1:1 for age, sex and ejection fraction on echocardiogram. We will include patients with atrial fibrillation or other cardiac dysrhythmias in association with the cardiomyopathy. Seropositivity for Chagas will be based on an ELISA test performed on every patient enrolled in the study. Exclusion criteria are designed to avoid confounding of the biomarkers or of the transcranial Doppler studies by major medical co-morbidities or anticoagulant therapy.

### Exclusion criteria

1. Patients with a history of an untreated malignancy (except local skin cancers),
2. Ischemic stroke (determined using the Questionnaire for Verifying Stroke-Free Status (QVSFS)),
3. Patients on renal dialysis or with end-stage hepatic dysfunction,
4. Acute infection/inflammation (Temp >101.5ºF, and/or WBC >15, 000).
5. Inability to obtain informed consent from patient or next-of-kin.
6. Anticoagulant use (warfarin or heparin).

**Cognitive evaluation.** Patients will be evaluated using a comprehensive battery of cognitive tests, including the Mini-Mental State Exam, Nitrini's Brief Cognitive Screening Battery [(includes the Clock Drawing Test, a series of immediate and delayed visual memory tests, and a verbal fluency test (animal sequence in 1 minute)]. Rey's Complex Figure Test will be used for evaluating visuo-spatial ability and visual memory. The Digit span test (forward and backward sequences) will be used for assessing attention and working memory.

**Biomarkers.** ELISA kits will be used for quantification of serum orosomucoid, neprilysin, IL-6 and MMP-9.

**Magnetic resonance imaging.** Magnetic resonance imaging (MRI) will be performed on a 1.5-Tesla GE scanner. Axial contiguous 5mm slices on T1, T2 and FLAIR sequences will be obtained for all patients. ImageJ software (freeware from NIH) will be used for volumetric analyses. Brain spectroscopy will be used to measure occipital lobe N-acetyl aspartate (NAA), choline (Cho), myo-inositol (MI) and creatine (Cr).

**Transcranial Doppler.** Transcranial Doppler (TCD) will be performed with a monitoring helmet. A single investigator will perform all tests, blinded to all clinical information. Vessels around the circle of Willis will be insonated through the temporal bone window. The right middle cerebral artery will then be monitored for 1 hour searching for high intensity transient signals (HITS). In the event of an absent right temporal bone window, the left middle cerebral artery will be located and monitored for 1 hour. Definition of HITS will follow previously published criteria, i.e., unidirectional transient signals >5dB.^81^

**Aspirin treatment.** Patients with HITS detected on TCD will be screened for exclusion criteria for aspirin use (bleeding disorders, alcohol use of 3 or more drinks per day, pregnancy in the third trimester, gastrointestinal symptoms or history of peptic ulcer disease, renal failure, severe hepatic insufficiency, hypersensitivity to NSAIDs, children or teenagers with chickenpox or flu symptoms, and syndrome of asthma, rhinitis or nasal polyps). Patients not fulfilling any of the exclusion criteria will be randomized in a 1:1 allocation to receive either 300 mg of aspirin or no treatment in an open-label fashion.

Current recruitment of the first 90 patients produced the following results presented at the World Stroke Congress in 2012:

Ninety patients were recruited, mean age 55±11 years, 51% female, 38 (42%) with Chagas disease (CD). Mean NAA/Cr ratio was lower in patients with CD as compared to other cardiomyopathies (1.73±0.17*vs.* 1.83±0.22, p=0.024), while MI/Cr and Cho/Cr ratios did not differ significantly. Long-term memory and the clock-drawing test were also significantly worse among CD patients. On the multivariable analysis, correcting for ejection fraction, age, sex and educational level, reduced NAA/Cr (p=0.006) and cognitive dysfunction (long-term memory, p=0.023; clock-drawing test, p=0.015) remained associated with CD. Based on these results, we conclude that Chagas disease is associated with cognitive dysfunction and decreased NAA/Cr independently of cardiac function or educational level.

The long-term goal of this project is to establish non-invasive methods of stroke risk stratification and prediction of stroke outcome in patients with Chagas disease. This work will also facilitate the development of novel anti-trypanosomal, anti-inflammatory, and antithrombotic strategies for stroke prevention and management in Brazil.
